# Consideration for Assessing Data/Models/Tools Expiration Supporting Drug Development and Clinical Decision Making

**DOI:** 10.1007/s43441-025-00793-z

**Published:** 2025-05-09

**Authors:** Jeffrey S. Barrett, Mark A. Turner

**Affiliations:** 1 Aridhia Digital Research Environment, Glasgow, UK; 2 Institute of Life Cycle and Medical Sciences, University of Liverpool, Liverpool, UK; 3 Conect4children Stichting, Utrecht, Netherlands; 4 Aridhia Bioinformatics, 163 Bath Street, Glasgow, G2 4SQ Scotland

**Keywords:** Data expiration, Clinical decision making, Drug development, Regulatory science

## Abstract

Decision making of any kind is informed by data and often by models, tools or other solutions built from data. Data are evaluated for such purposes within a specific context of use (COU) but implicitly we often believe the data to be relevant, accurate and of high quality. In reality, this is not always the case. The status of data for various COUs must constantly be revisited for relevance and information value over time. Using drug development as an example, we postulate that there are indeed occasions where data value diminishes over time and consideration for data expiration with respect to its relevance for decision making should be entertained and at least identified with respect to a time-dependent change in status. Other situations exist which will also necessitate periodic review and condition reassessment. For example, considerations for patient privacy and consent along with compliance to regulatory standards must factor into future recommendations as well. Actions regarding data expiration are proposed as initial thoughts to be expanded upon but this assessment is primarily an attempt to explore factors which impact opinions about data information value for both drug development and clinical decision making.

## Introduction

A commercially-available medicine is typically labeled by the manufacturer with an expiration date. This reflects the time period during which the product is expected to remain stable, or retain its identity, strength, quality, and purity, when it is properly stored according to its labeled storage conditions. In the case of a medical product or clinical samples, expiration may result in the disposal or elimination of the material in question [1]. Such rules with respect to data do not exist with the same clarity. Data may be praised and valued as an asset across multiple industries (e.g. financial and retail industries); however, it also has an expiration date—one based typically on the speed at which business currently occurs. Consider the situation of on-line credit card transactions (validity of card number and account details) and loan decisions in the banking industry (credit scores, financial viability, etc.) (Shivram 2024 and [2]. Both decisions have different time scales relative to their business practices. Many other data-centric sectors are well familiar with the concept of data stagnation which calls into question considerations of data expiration. For instance, even the basic information such as phone numbers and email addresses change constantly over time. Likewise, data can become outdated, incorrect, or inapplicable over time. However, data expiration does not have to mean data deletion. For example, data on the natural history of a disease may change because a new treatment becomes available. This does not mean that the natural history prior to the development of the treatment is not useful but it should be reviewed with caution because new data are available that changes the relevance of the older data.

The concept of data expiration from a data management perspective is understood as the date at which a record is eligible for archiving or deletion. The expiration date equals the retention period plus a date that is determined by the retention policy rules. Execution rules and the context for expiration are typically the issue requiring careful consideration [3] before policy can be implemented. There is limited experience in these issues but the existing policies are not really portable to drug development or clinical decision making.

Data expiration policies in general [4] are well appreciated but these often refer to both currency and value–when data no longer represent recent data of interest. Currency of data is related to how the data reflects the condition(s) of interest. If conditions change (new information, new practice, etc.) certain fields collected in a dataset may become nonrelevant and lose value for decision making [5]. For drug developers the same is true although there is commonly not an articulated policy around which data are susceptible for these conditions and likewise deletion and under what circumstances are they no longer informative or valuable.

There is likely some sensitivity around the term deletion with regards to data. Regulators may need to check the analyses and data that contribute to regulatory decisions. For this reason, regulators such as the European Medicines Agency (EMA) have put forward concepts such as ‘data immutability’ [6]. The EMA definition of data immutability includes “Data immutability is the concept that data is never deleted or altered. Once some data is “stated” (e.g.: entered in a database), it can only be augmented (eventually with additional information meant to invalidate or supersede previous data) but never remoted. In other words, data that have been entered in a system (and on which some other data or actions may depend) cannot be changed without explicitly mentioning of a new state of the information and maintaining the knowledge of the previous state.” As an example data generated using an assay or technique which has improved or evolved over time making the old assay results less reliable or informative may necessitate that the old data (and results) not be used as an anchor for comparison when decision making is critical (e.g. bioavailability or extrapolation). It should be understood then that “expiration” or an expired status for specific data refers to the utility of the data to make good decision and the conditions by which data status changes with respect to its information value for specific contexts of use.

Likewise, health care providers may need to assess secondary analysis of data to check how medicines are used in clinical practice. This includes foundational data. One example is the derivation of a lifetime approach to glomerular filtration rate using data gathered in multiple studies over more than two decades [7]. Such data is used to assess drug utilization correlated with clinical outcomes as the basis for both formulary and reimbursement decisions. Another foundational example for observational data is the data about birthweight and health outcomes in later life which was based on meticulous record keeping in routine practice in the north of England.

Data owners or assemblers of data contributed by patients need to protect patients’ rights by only allowing access and use based on the original informed consent and need to be able to manage data status and access in those situations where individuals’ rescind their consent [8]. Organizations that promote the contribution of patient data so that patients can share among themselves or provide their data in the hope that others will utilize the information to accelerate the development of new treatments and possibly cures are likewise expected to routinely evaluate the contributed data over time and modify what is collected as knowledge about disease biology and clinical science evolves (e.g., NORD (National Organization for Rare Disorders), Rare X and the Critical Path Institute) [9].

Recognizing that data generated and/or acquired during drug development has different purposes and a variety of contextual uses, we have attempted to assess this issue from various perspectives and created a position from which future discussions will hopefully lead to a defendable default policy for various stakeholders. We review the underlying source data used at various stages of drug development along with the complementary models developed to inform decision making and common use cases. We similarly explore and discuss data elements and tools used for clinical decision making. We propose a plan of status management for clinical data across its lifetime. We identify several points to consider when developing a data lifetime management plan. Similar considerations apply to preclinical data but are not addressed herein.

### Data Generation in the Context of Drug Development

The commonly appreciated stages of drug development include discovery, product characterization, formulation development, delivery, packaging development, pharmacokinetics and drug disposition, preclinical toxicology evaluation, additional preclinical evaluation leading to an investigational new drug (IND) application in the US (or equivalent regulatory document elsewhere), bioanalytical testing, clinical development including clinical trials culminating in a regulatory submission (NDA for the US FDA) if successful. These stages are distinct with some overlap and generally but not entirely sequential. After passing preclinical safety and proof of concept (POC) hurdles, a molecule ready for development could be worth many times the cost of the safety /toxicology investment (or not). The return on investment through this step can be quite high for those who can take the risk [10]. There is a financial incentive for companies to adopt a “quick kill” approach where they adhere to Go/No-go criteria and define quantitative stage gates by which they will judge the progress of development of a drug candidate. In this way, the attributes that the project team and senior leadership have agreed to will be required to be met or surpassed or the compound will be removed from development consideration. The sooner this can be determined in the development phase, the better from a financial point of view, hence the “quick kill” designation. Data are necessarily generated by multidisciplinary functional groups to support decision making and, assuming success, represent the proof that a candidate is suitable for human phase testing (IND) or market approval (NDA).

Historically and until recently such data were maintained in documents with the official source being paper copy [11]. As database technology matured and the movement towards electronic regulatory submissions ensued [12, 13] the value in maintaining internal data sources became more appreciated and investment into electronic data sources and archival was initiated. An unfortunate habit of the early practice was to maintain data for active or approved compounds while discarding failed or quick-killed compound data. The practice diminishes the value of such accumulated data [14] as it is biased by successful outcomes. Still, it began a situation where pharmaceutical sponsors had to invest in data storage to maintain data on active candidates in addition to approved products where the historical querying of such data might be warranted.

In addition, while the majority of drug development data is generated by the sponsor or for the sponsor by contract research organizations (CROs) there is the growing trend to acquire external data for various decision-making purposes. The type of data acquired and intended context of use (COU) varies dramatically of course. Acquired data could include chemical libraries, formulation excipient databases, natural history data, claims data, electronic medical records, patent data and other data sources serving both R&D and commercial interests. It can also vary based on size, format making integration with in-house data a challenge. Each of these sources should be identified via meta-data catalogue with respect to origin and audit history. Likewise, one cannot assume that just because a drug developer pays for data that it is of adequate quality and meets requirements for data standards and integration.

### Assets Built from Data

Another component of drug development decision making is the generation of models, algorithms and tools that further aid executives in the process but that are also constructed from or informed by data. These include but are not limited to PK/PD (pharmacokinetic-pharmacodynamic), PBPK (physiologically-based pharmacokinetic), QSP (quantitative systems pharmacology), DP (disease progression) and CTS (clinical trial simulation) models along with coding and simulations that support and qualify the models respectively. As with data, consideration for expiration dates or management at different stage of an asset’s lifetime for such assets need to be considered, particularly if there is a cost to maintain them with a certain expectation on value and currency. Table 1 provides some initial considerations on both data and asset lifetimes. One important consideration is the assays that are used to measure drug concentrations or endpoints. It is natural for assays to evolve (i.e., become better due to improved sensitivity, specificity or accuracy). In addition, assays may become unavailable because materials or machines become replaced or modified or expertise is lost. This essentially creates confounding with the data generated by specific assays requiring the user of the data to assess if data generated by different assays is reporting similarly (i.e., can the data be pooled across various assays).

**Table 1:** Data types supporting drug development decision making, assets derived from data and COU-supported initial thoughts regarding expiration

Data type/format	Stage(s) of development	Assets derived from data	Considerations for expiration
Company generated			
Physiochemical data, in vitro screening data, in silico ADME data	Discovery, preclinical evaluation	In silico ADME, PBPK and QSP models	Molecular characteristics don’t generally expire and are useful as anchors for predictive models and AI/ML approaches. Some consideration might be given to data generated by old or now outdated methods, outdated assays or where experimental conditions were suspect
In vivo safety and pharmacology data, formulation study data		PBPK and QSP models	Potential for analytical issues
Healthy volunteer PK/PD	Phase I	PK and PK/PD models	Analytical issues or concerns that PD was not relevant^1^
Patient PK/PD	Phase II and III	PK and PK/PD and CTS models	Analytical issues or concerns that PD was not relevant^1^
Acquired external (purchased or attained via agreement)			
RWD (EHR data)	Phase II–IV	PK and PK/PD, DP and CTS models	SOC considerations
RWD (Registry and natural history data)	Phase II–IV	PK and PK/PD, DP and CTS models	Biomarker or SOC considerations
RWD (claims data)	Post-marketing	HECON and CTS models	SOC or marketplace considerations
Surveillance data	Pre or post marketing	HECON, DP and CTS models	Poor sampling, Biomarker or SOC considerations

*ADME* absorption, distribution, metabolism and elimination; *PBPK* physiologically-based pharmacokinetic; *AI/ML* artificial intelligence / machine learning; *QSP* quantitative systems pharmacology; *SOC* standard of care; *HECON* health economics; *CTS* clinical trial simulation; *DP* disease progression; *RWD* real-world data; LOD = limit of detection; *EHR* electronic health records

^1^For example, in the case where the assay LOD was insufficient to accurately assess the PK or the PK was incorrect because the assay did not capture the terminal phase

Regardless of the source of the data or the intended use (drug development or regulatory decision making or clinical decision making) data status can change over time due to either intrinsic or contextual considerations that can change over time. Table 2 provides a description with examples for each category.

**Table 2:** Intrinsic and contextual rationale and examples for changing data status over time that may influence considerations regarding data expiration

	Contextual	Intrinsic
Meaning / description	Related to the use or context of data application with respect to decision making	Related to the inherent nature of the data and implicit to how it was generated, collected or defined
Examples	• Clinical data which no longer reflects the standard of care (time-dependent change) for the intention of a comparative treatment as with a clinical trial simulation • Data (preclinical or clinical) used for pediatric extrapolation when the diseases are found to be dissimilar • Clinical markers initially thought to be surrogates (e.g., ACE inhibition, CD4 counts, etc.)–time dependent recognition of biomarker’s value and/or suitability as a surrogate endpoint	• Poorly collected (inappropriately sampled) concentration–time data for PK analysis • Measurement of GFR to assess renal status and kidney function • Assessment of liver enzymes, particularly the boundaries of the upper limit (UL) as clinical trial exclusion criteria

One of the concerns regarding the currency and utility of models used for decision-making is the reliance of models constructed on old or “stale” data. Ironically, the concept of data staleness and “data drift” impact is more commonly understood for more recent application of AI [15] and/or machine learning models which are more transparently understood as impacted by data quality although the same is true for any model constructed from data [16]. It should also be apparent that the sensitivity of the modelling type or approach to data staleness, data quality and information value is linked to the COU.

Related to this issue is the vulnerability of certain models and model complexity to data condition. Some recent work also suggests that data type (e.g. imaging data) pose additional considerations on this issue [17]. Likewise for regulatory decision making linked to the review and approval of new drug candidates, the underlying data from which clinical trial simulation models are constructed must reflect both the current standard of care and the current knowledge on the specific disease progression of the target indication being clinically evaluated. Precision medicine strategies (tailored disease prevention and treatment) reliant on various data sources must also be well thought out with respect to identify the appropriate data elements or data transformations linked to improved clinical outcomes. Strategies to challenge the underlying assumptions periodically must be developed and deployed in order to ensure that predictive power is maintained. Typical assumption testing strategies would include tools and practices such as SWOT (strengths, weaknesses, opportunities and threats) analysis, scenario testing and planning (typically via simulations) and sensitivity analysis.

The nature of models utilized for various COU may create different vulnerabilities with respect to the underlying data (e.g., empiric versus mechanistic or simple versus complex) [18–20]. This can be appreciated in the context of sampling design. If sampling post dosing does not include early time points, the model can hardly support parameters describing drug absorption, especially those of a mechanistic or complex nature. Likewise, if a population is appropriately diverse covariates such as race or ethnicity may be impossible to evaluate or include in a model.

### Regulatory Perspective on Data Currency and Expiration

Regulatory authorities are continually engaged in the review and evaluation of sponsor’s data in the context of evidence of safety and efficacy required to support the registration and commercialization of new medicines. Evidence takes the form of data, both individual and summarized. Well defined standards for approval guide reviewers in this task. Likewise, sponsors have been given clear guidance that has evolved over time with respect to the data submitted to regulatory authorities. While they may be some subtle differences in global regulatory authorities requirements, for the most part there is excellent harmonization that facilitates a common approach for sponsors.

It is clear from a regulatory perspective that an element of trust in both the relevance and quality of data submitted by sponsors is expected [21]. Trust also extends to the models and tools constructed from data. Likewise, regulators have the expectation that sponsors understand and comply with the standards for approval. There is also the expectation of transparency around changes in plans for the collection of data, inspections and data integrity. Regulators attempt to guide this process and facilitate transparency via a number of mechanisms. The following example illustrates some of the historical efforts to instill trust and jointly develop approaches and mechanisms to leverage adult experience (e.g., data, models and tools) to improve confidence in decision making pertaining to pediatric drug development.

### Regulatory Example: Context of Pediatric Drug Development Considerations

The era of pediatrics therapeutics development is relatively new. Prior to the passage of important laws in the U.S. in the late 1990’s and early 2000’s, and in the EU in 2007, over 80% of drugs used in children did not have adequate information to support safe and effective use in product labeling. Since the passage of these laws, pediatric is now available for most prescription drugs. In fact, in 2022, the 1000th labeling change with pediatric-specific information was approved in the U.S.

During this time, many advances have been made to improve the efficiency and success of pediatric therapeutics development. One area of particular importance is in the use of pediatric extrapolation. Pediatric extrapolation has been defined as, “an approach to providing evidence in support of effective and safe use of drugs in the pediatric population when it can be assumed that the course of the disease1 and the expected response to a medicinal product would be sufficiently similar in the pediatric (target) and reference (adult or other pediatric) population (ICH E11A 2024).” Pediatric extrapolation, when appropriately applied and scientifically justified, can ensure that children only participate in clinical trials necessary to further scientific understanding of a drug’s use in children. Pediatric extrapolation can also increase the efficiency and success of therapies that have benefits in children.

Importantly, a fundamental principle of pediatric extrapolation is that evidence is available that supports that the disease, drug pharmacology, and response to treatment are similar between a reference and target population. The degree to which these elements are similar between a reference and target population are based on a review of all the relevant data. Data relevance can be directly impacted by the “freshness” or “staleness” of the source and types of data reviewed. For example, an important element in understanding similarity of disease between a reference and targe population would be to compare the natural history of the disease between the two populations. Natural history data often are derived from real world data sources (e.g., patient registry data, observational studies, and case reports/medical chart reviews. If a treatment becomes available in either the reference or target population, the availability of treatment can potentially change the natural history of the diseases. If this occurs, data on natural history of the disease before the availability of the approved treatment would likely become stale (i.e., less relevant), and such data may no longer be useful in supporting similarity of disease. Thus, natural history data, even if carefully collected, may become “stale” and keeping track of the age of the data with respect to its use in supporting a pediatric extrapolation approach is of clear importance. Stale is this context would refer to the currency of data for a particular context of use. Likewise the detection of data for a particular COU would likely involve searching for fields or identifiers related to an indication of “staleness.” For instance, if a certain biomarker or lab value was collected for years as a marker of disease progression and then was replaced by a new assay or test with better predictive value, continuing to record the old marker may be less important if it is no longer used in the same manner, especially if it has been replaced. Failure to keep track of the relevance of the data could lead to incorrect conclusions about the similarity of disease between a reference and target population and could lead to false reliance on extrapolation. The end result could be to approve a drug in a pediatric population that is actually ineffective or unsafe (e.g., KIDS list) [22]. Clearly this situation should always be avoided.

Another area where the relevance of the data are crucial in making accurate predictions is in the use of model informed approaches during pediatric drug development. Modeling has been used to help understand all aspects of pediatric trials, from dose selection to evaluation of data to support a pediatric extrapolation concept, to development of appropriate pediatric study designs. PBPK, QSP, and other models can be developed based on use of data from many sources. Again, failure to identify data that are relevant and up-to-date could lead to false assumptions in the developed models. This in turn would lead to incorrect conclusions about the validity of the model used. This sentiment has been articulated in the recent ICH guidance on pediatric extrapolation [23]. Ultimately, the success of the model depends on the quality and relevance of the data used to develop the model to avoid incorrect conclusions drawn based on model-outputs.

Other initiatives outside the general drug development process include the recent considerations for RWD (real world data) and RWE (real world evidence) to complement and support randomized control trials (RCTs) in the demonstration of safety and efficacy in patients and considerations for drug repurposing where data may be borrowed from other indications or past development activities. Reliance on real world data when running a clinical trial must also contain an assessment of any time dependencies that could implicate certain time domains if such changes create uncertainty in information value and utility for decision making. Particularly, if electronic health records are used as RWD sources, pulling across systems (e.g., EPIC versus Cerner or others) may present challenges as well. In addition, in the area of rare diseases, patient advocates must understand that some data are not necessary to keep and may not be valuable. On the topic of drug repurposing sponsors must appreciate the context of data to support an approval of a drug long ago may not be sufficient to support the approval for a new indication in a new context. Likewise, RWE if it is to substitute or complement an agreed-upon clinical endpoint from a RCT must carry the appropriate level of confidence from a data quality perspective as communicated clearly by global regulatory authorities.

### Clinical Assessment of Data Utility in the Context of Decision Making

Clinical data of various kinds are used routinely to make important decisions with respect to patient diagnosis, treatment options and specific treatment management (e.g., dosing of medicines and duration of therapy). Likewise, there are predictive models and decision support systems that are constructed from such data that also contribute to clinical decision making.

Of course the data types and sources for clinical decision making are different than those used in a drug development but they carry the same concerns with respect to uncertainty, quality and information value as discussed previously. As with drug development there are varied data generators, owners, repositories and structures. While many data types are consumed with hospital-based electronic medical record systems, many are not. Very few centralized systems exist although there are great strides being made at some institutions, particularly those engaged in research [24] in addition to patient care [25–27].

In order to identify how data status and clinical decision making impact on each other we need to review briefly clinical decision making. Ideally, clinical decision making about using a therapy is linear, rational, and based on complete information (where information is a concept that includes data, evidence, guidance, and clinical circumstances). In practice, the pathway from data to decision is not straightforward. Multiple systems interact. This includes physiological systems so that, for example, PKPD relationships may not hold in the presence of co-morbidities. Data may be “true” but not useful. Social systems can also lead to unpredictable events such as the relationships between regulatory decisions, health care reimbursement decisions, and preferences of people living with conditions. Data may be “true” but used in different ways for different purposes. “Incorrect” data may be neutral, without influence, or be an approximation to the truth that is better than no information, or be misleading. Furthermore, there is usually some uncertainty about the individual and about the properties of the therapy in the specific context of the individual. In the real world, any data is better than no data as long as the relevance and quality of the data is clear.

Likewise, models and tools constructed from data used in clinical decision making, such as Clinical Decision Support Systems (CDSS) carry both data quality expectations [28] along with requirements around the predictive performance of the tool in addition to some form of clinical validation [29–31]. CDSS have been for many purposes including diagnosis, triage in emergency settings, medication management, and image analysis. While there are many examples of models used to provide patient diagnosis, guide treatment options or assess the performance of clinical interventions [32, 33], (Freedman 2001 and Hann 2005), most of the published description is based on model or tool development and testing than about their longitudinal performance or reevaluation over time. Data expiry is relevant to computer CDSS, for example as coding data [34] or clinical practice change [35]. It is good practice to update decision tools [30, 31, 36–39]. In passing, we note that updating and revalidating CDSS are not included in the relevant FDA guideline [29].

Model creep (shift in the model appropriateness for a given COU) is a risk if a CDSS is used widely and the stewards of CDSS need to ensure the model is used in ways that are compatible with its initial validation. This assurance will benefit from access to the data used to train and validate the model. Model creep can also occur with time. For example, the standard of care inherent in the context of use for which a CDSS is validated may change and this change may or may not affect the accuracy of the CDSS. Data simulations may be useful to check the boundaries of accuracy in which case the initial data for validation needs to continue to be accessible to the developers and reviewers of CDSS for as long as the CDSS is in use.

If CDSS are involved in unwanted outcomes, CDSS could be included in litigation. It is conceivable that litigation would involve some assessment of whether the CDSS was fit for the use that led to the unwanted outcome. That assessment could require re-evaluation of the initial validation and subsequent application to a specific use case. The risk of litigation could be mitigated by retaining access to data used to teach and validate CDSS until the relevant statute of limitations has passed.

**Figure 1:**
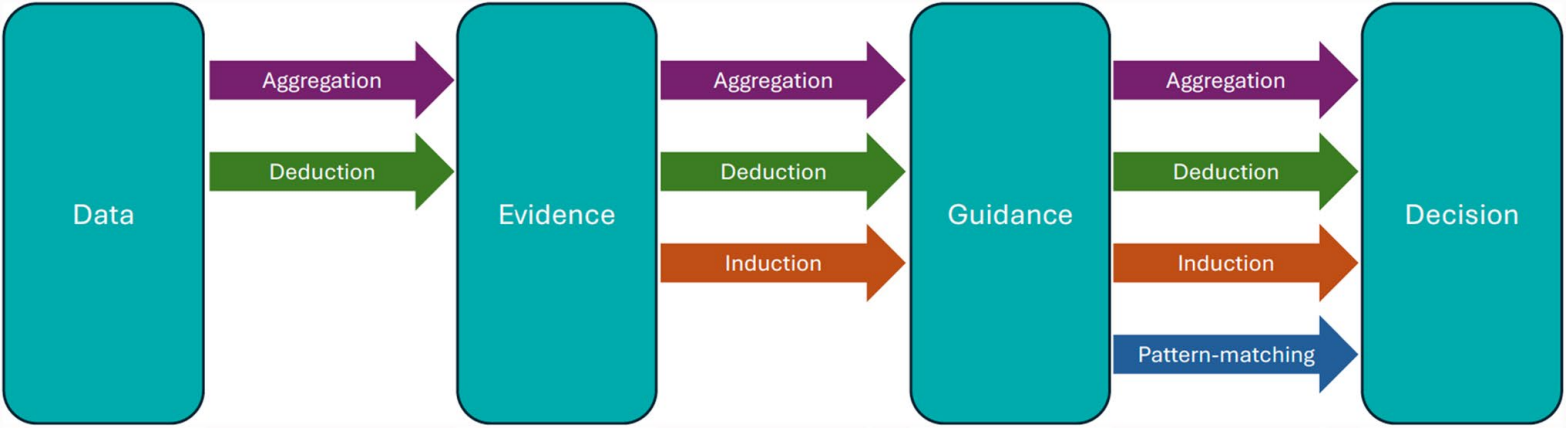
Standard pathway from data to clinical decision-making. Data refers to information gathered during research. Evidence refers to curated data. Guidance refers to evidence presented to support decision-making (this is a combination of explicit guidance among health care professionals, implicit frameworks such as personal algorithms based on experience, and decision support tools). Decision refers to the process of selecting a course of action by synthesizing guidance with the information relating to the specific circumstances of a clinical case. Aggregation includes means of groups, single study conclusions, conclusions from multiple studies. Deduction refers to applying data or evidence within the study population(s). Inference refers to applying data or evidence beyond the study population(s)–generalizability. Pattern-matching refers to the generation and deployment of consistent relationships between information.

A generalized pathway from data to clinical decision making (computer supported or not) is shown in Fig. 1. Most clinical decisions do not use primary data but are based on guidelines from professional groups or heuristics derived from personal experience. Guidelines are informed by Evidence generated from Data during drug development. Evidence includes drug labels (SmPCs or Summary of Product Characteristic) in the regulated space or other forms such as Cochrane Reviews in clinical practice. It may be difficult to separate the salience of one piece of data to guidance or decisions. The development of guidance often involves comparisons and trade-offs based on how information is weighted compared to other information. Even if a data point changes it may leave a memory or ghost caused by the way it influenced the weighting of other information. In the physical world we have quantum, Newtonian, and systems pharmacology effects which are different types of effect and difficult to map between because of scale. Something similar can occur in pathways from data to clinical decision. A change at one step of the pathway may not have identifiable effects at another step.

Ideally data needs to be accessible through the sequence from data to decision. The status with respect to accessibility is part of the metadata for each datapoint. We need to consider the implications of data status on subsequent stages in the sequence: traceability; nature of imperfections; relevance. If data expires, it isn’t necessarily replaced–poor quality data may be better than no data–prejudices and inferences live on. This view of the relationship between data and decisions is not an argument to keep everything “just in case” but identifies some points to consider when reviewing data status. We need to consider backwards (from bedside to bench) implications on data status as well as forwards (bench to bedside).

Model-informed precision dosing (MIPD) can use primary data from drug development directly in decision making so that changes in data status need to be transmitted to all users. Moreover, Model-informed precision dosing may need access to unaggregated data which may represent a challenge in both the operational timing and the update of the analytics producing the dosing recommendation. The effort required to keep MIPD and decision-support tools updated and continuously validated may be considerable. There is a risk that poorly supervised and unregulated models could lead to unwanted events arising from medication use–a virtual thalidomide crisis with real-world implications. A necessary, but not sufficient, condition for optimal supervision is clarity about which data underpins models. Ideally, this is based on deep Open Access to data sources [40] rather than superficial versions of Open Access such as “Open Weighting” [41].

Patient advocates expect health care research data to be shared when that data could help other people. Unjustified deletion or making data inaccessible will conflict with the altruism that stimulates many participants in health care research. Deletion or making data inaccessible needs consent/assent bearing in mind the “right to be forgotten” that prevails in some jurisdictions. There are difficulties in giving fair access to consent/assent particularly as young people attain majority. One option is to include a clear statement that data will only be held for so long. There is a need to plan the ideal prospective use of with appropriate consent/assent for expiry and how to work with existing data with incomplete consent/assent.

As long as data is annotated as well as practicable it can be retained somewhere for potential secondary use or recognition of contribution to sequence of data use that supports clinical decision making.

There are many points to consider when annotating data and deciding on its level of visibility. A repository of record is needed, with a baseline of annotation and visibility. Other storage facilities may need clear policies and a plan for each dataset. Lack of annotation may not prevent all data reuse. The tolerable level of precision depends on the context of use. The ‘‘risk terrain” influences tolerance. In the context of risk/benefit, therapeutic index will guide the extent to which imprecision can be tolerated. With respect to content details of specificity and overlap will be beneficial. There are some practicalities that will need to be addressed, namely technical/semantic interoperability. Likewise, planning for post hoc annotation is reasonable. Finally, a mechanism to deal with and track the management of errors should be considered along with control and access of data.

An easy default position to take is to not consider expiration of data in any meaningful context. Deletion would only be considered if there was no foreseeable use or the data is just too expensive to retain. However, it is probably more informative to entertain what is reasonable.

### Setting Realistic Expiration Dates for Data–What’s Reasonable?

While simplicity is often desired in any setting requiring rule-based decision making, it is often unattainable. Setting “expiration dates for data” likely falls in that category and it may be better to think about conditions around data and data use that necessitate action of some kind by the data owner, curator or user. A contributing factor for this situation is the varied tolerance and risk-taking based on uncertainty across the diverse stakeholders on both the regulatory and clinical settings [42]. Consider the attention to the development of clinical decision support software and regulatory oversight implied based on FDA guidance [FDA Guidance, date]. Most of the emphasis pertains to verification and validation of the underlying algorithms and tools and not the underlying data or data processes. Part of the intention of this communication is to draw attention to this oversight and expose the underlying concerns.

Both data type and COU dependency are relevant of course. Criteria (quality, currency, information value, etc.) for assessment are obvious but considerations on frequency of checking also need to be viewed under the circumstances of the decision risk and criticality. The frequency of reasonable data quality checking ensures consistency in the interpretation and decisions derived from data. Commercial data quality monitoring solutions exist for real-time assessment; for example, IBM Databand connects data pipelines and datasets to alert on problems like schema changes, duplicates, null values and data freshness and provides a means to visualize datasets over time but cost is also likely a consideration for many organizations.

## Discussion

The topics of data quality, data value, stagnation and expiration are intimately linked with the understanding that data is a key component of both drug development and clinical decision making. Understanding the landscape for decision making from the standpoint of data requirements (quality, currency and information value) is an essential step toward making any recommendations for data policies and must be indexed up on various contexts of use (COU). Data quality considerations are diverse and complex. The adequacy of data is also an important aspect of quality. For example, are baseline variables defined in a clinical or disease process sufficient to describe the patient, their interventions, outcomes, etc. in sufficient detail? Considerations on the potential for selection bias or misclassification may also be relevant. Other questions related to the underlying data and data processes must also be answered. Size? Speed of data availability? Safety monitoring requirements? Changes in collection and recording practice? Patient follow up? Differences in health systems? Validated linkage of data?

We’ve highlighted the data elements used for decision making at various stages of drug development along with the data types often used in clinical decision making which are constantly growing. We’ve also explored the various model types used to facilitate decision making in both settings. In the end our primary recommendation is to assess data quality, currency and information value periodically with the intention to flag the status of each attribute dynamically for various COU. Similarly, models and tools reliant on data can be similarly flagged so that recommendations generated from such tools can be viewed with the relevant caveats.

It may be impossible to define rules for data expiry in all situations. We advocate for considering expiry through an explicit framework for decision-making about data status that would allow consistency, transparency, and provide information needed for trade-offs, e.g. between costs and universal access. The components of a framework to support decision-making about expiry or changes in the extent of accessibility can include a variety of factors. We’ve outlined some initial considerations in Table 3 below.

**Table 3:** Initial considerations for a framework of key factors supporting decision-making about data expiration

Factor	Drug development concern(s)	Clinical concern(s)
Nature of data including quality	• Will need to consider stage of development and “risk assessment” related to the decision	• Risk assessment related to the impact of the decision (choice of drug, dose, etc.)
Current access to data	How and who? Costs to user and costs to host	
Extent data annotation	• Later stages of drug development benefit from audit trails to be shared with regulatory authorities • Ownership, dimensions, quality assessment, etc. indexed on COU	• PII considerations, methods and methodologies • Quality and risk linked to clinical decisions
Data use purpose	• Relevant to stage and quality considerations	• Patient privacy, PII in general may need consideration
Contexts of use relevance	• Relevant to decision-making milestone; may have regulatory implication	• Linked to clinical decision; will need to be annexed to patient outcomes
Data state types	• Format, structured or unstructured and details of standards (e.g. CDISC)	• Data origin (e.g., EHR) and format considerations linked to required transfer or transformation
Consequences of change in data state	• QA considerations on “final” dataset; regulatory implications	• Inability to revalidate CDSS or check validity of labelling
Consequences of not changing data state	• Audit trail, SOP and regulatory implications	• Cost, predictability, information value
Options appraisal	Do nothing, change data state of some or all of the data	
Decision	• Allow traceable decision trail linking data, models and tools to milestone decisions with a COU	• Create a traceable pathway of clinical decisions within patient and across relevant patient groups

One of the obvious recommendations from this assessment is the necessity of periodic review of data in both drug development and clinical domains. Metrics to assess quality, currency and information value linked to various COU are highly desirable for this review. Most importantly, being able to implement such reviews in a scheduled and automated manner is also highly desirable. Technologies to help automate the assessment of data quality in general with an opportunity to assess utility (staleness) for certain COU with rule-based checks evaluated over time are few but there are several which offer promise. One of the more intriguing options is to exploit the potential of bot technology to automate the data assessment process and generate on-the-fly data currency / value metrics. Data simulations could be used to identify potentially critical data or which changes in data can be tolerated.

Bots (autonomous programs on the internet or another network that can interact with systems or users) are core to broad functioning of the internet. While they make our lives easier in ways that aren’t always obvious, there are bad bots too, and they infest nearly 28% of all website traffic. From spam, account takeovers, scraping of personal information and malware, it’s typically how bots are deployed by people that separates good from bad [43]. Generative AI like ChatGPT make it harder to discern the distinction between bots end humans. The use of bot technology to assess data quality is an intriguing use for good that could be further developed to aid assessments for proposing expiration dates as well. Rather than relying solely on manual intervention, teams can ensure quality by creating a scoring system through which they identify common bot tactics. A nice example of the positive utilization of bots is the deployment of Archie, Eureka™ and BB bots by the Data.World organization and their Data Catalogue platform (https://data.world/). Collectively, these bots reduce the manual human effort required to find and understand data with curations powered by large language models (LLMs) like OpenAI’s GPT, eliminate tedious, manual data work, and helping governance teams automate metadata enrichment, access workflows, and agile processes and improve efficiency by creating seamless communication between data teams and data consumers on data quality and usage.

Building a measure of quality requires subjectivity to accomplish. Researchers can set guardrails for responses across factors. While we need to be skeptical of data and build systems to standardize quality, applying a point system to assess these traits, researchers can compile a composite score and eliminate low-quality data before it moves on to the next layer of checks and become a part of data used for decision making or in tools that further inform decision making.

## Conclusions

Moving forward for both clinical and regulatory decision makers, it would seem obvious that we need to define data quality rules and metrics to be tracked and alerted with a predefined frequency. Coincidentally, decision makers should identify and align observability tools to overall data governance and management strategy. Preparation for any potential process changes and role shifts based on the outcome of each periodic assessment would be prudent. Partnering with relevant stakeholders, the data engineering team, the data quality team and financial governance experts to evaluate and demonstrate business value of data observation practices is always a good idea. A recent report from Gartner [44] highlights this issue with more granularity and discusses the landscape of technologies currently available to address data observability and quality. The issue of information value tied to COU is more difficult and will require more contribution from the stakeholder community especially considering the varying degree of risk tolerance likely.

## Data Availability

Data generated herein for this analysis is based on literature and web-review; links to open source solutions, works in progress and use cases are provided in the text.

## Declarations

### Principal Investigator’s Statement

The authors confirm that Jeffrey S. Barrett, PhD, FCP is the PI responsible for this research and analysis.
